# 2-Fluoro-l-histidine

**DOI:** 10.1107/S1600536810038663

**Published:** 2010-10-02

**Authors:** Kiran K. Andra, John C. Bullinger, James G. Bann, David M. Eichhorn

**Affiliations:** a Department of Chemistry, Wichita State University, 1845 Fairmount, Wichita, KS 67260-0051, USA

## Abstract

The title compound, C_6_H_8_FN_3_O_2_, an analog of histidine, shows a reduced side-chain p*K_a_* (*ca* 1). The title structure exhibits a shortening of the bond between the proximal ring N atom and the F-substituted ring C atom, indicating an increase in π-bond character due to an inductive effect of fluorine.

## Related literature

For the structure of l-histidine, see Madden, *et al.* (1972 ▶). For the use of 2-fluoro-l-histidine in biochemistry, see Eichler *et al.* (2005 ▶); Wimalasena *et al.* (2007 ▶). For a related synthetic procedure, see DeClerq *et al.* (1978 ▶).
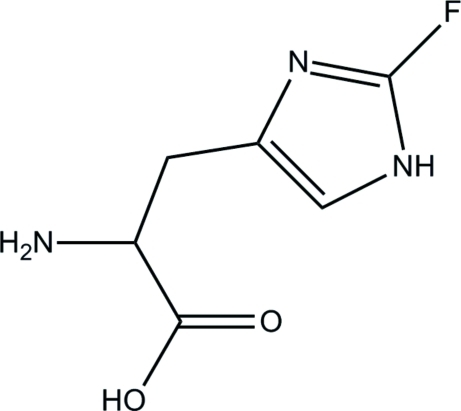


## Experimental

### 

#### Crystal data

C_6_H_8_FN_3_O_2_
*M* *_r_* = 173.15
Orthorhombic, 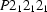

*a* = 5.1880 (3) Å
*b* = 7.3480 (5) Å
*c* = 18.7169 (12) Å
*V* = 713.51 (8) Å^3^
*Z* = 4
Mo *K*α radiation
μ = 0.14 mm^−1^
*T* = 150 K
0.16 × 0.14 × 0.13 mm

#### Data collection

Bruker APEXII CCD area-detector diffractometer
Absorption correction: numerical (*SADABS*; Sheldrick, 2000 ▶) *T*_min_ = 0.978, *T*_max_ = 0.983
3663 measured reflections
1352 independent reflections
1257 reflections with *I* > 2σ(*I*)
*R*_int_ = 0.022

#### Refinement

*R*[*F* ^2^ > 2σ(*F* ^2^)] = 0.043
*wR*(*F* ^2^) = 0.125
*S* = 1.06
1352 reflections
109 parameters
H-atom parameters constrained
Δρ_max_ = 0.42 e Å^−3^
Δρ_min_ = −0.47 e Å^−3^

### 

Data collection: *APEX2* (Bruker, 2005 ▶); cell refinement: *SAINT* (Bruker, 1996 ▶); data reduction: *SAINT*; program(s) used to solve structure: *SHELXS97* (Sheldrick, 2008 ▶); program(s) used to refine structure: *SHELXL97* (Sheldrick, 2008 ▶); molecular graphics: *Mercury* (Version 2.3; CCDC, 2009 ▶); software used to prepare material for publication: *SHELXTL* (Sheldrick, 2008 ▶).

## Supplementary Material

Crystal structure: contains datablocks I, global. DOI: 10.1107/S1600536810038663/im2231sup1.cif

Structure factors: contains datablocks I. DOI: 10.1107/S1600536810038663/im2231Isup2.hkl

Additional supplementary materials:  crystallographic information; 3D view; checkCIF report

## Figures and Tables

**Fig. 1.**
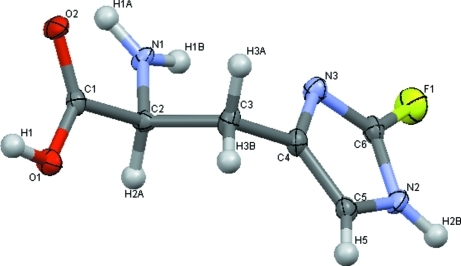
Mercury plot showing thermal ellipsoids on the 50% probability level.

**Fig. 2.**
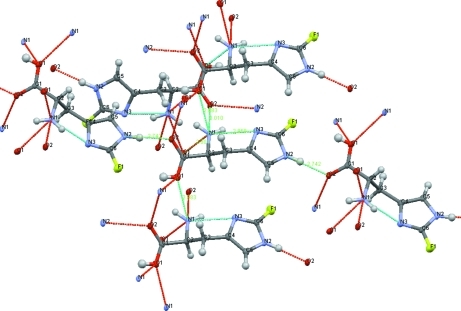
Mercury plot showing the hydrogen bonding network.

## supplementary crystallographic information

### Comment

We have investigated the structure of 2-fluoro-L-histidine (2-FHis) by single-crystal X-ray crystallography. The objective is to utilize this structure for future use in determining protein crystal structures which incorporate this unnatural amino acid. An isosteric analog of histidine, 2-FHis has a greatly reduced side-chain p*K*_a_, on the order of 1, and can be used to probe the role of histidine in enzyme mechanisms or biomolecular interactions (Eichler *et al.*, 2005; Wimalasena *et al.*, 2007). The present crystal structure is similar to L-histidine (Madden *et al.*, 1972), but with distinct differences that are certainly due to an inductive effect of the fluorine. The fluorine atom substituted at C-2 of the imidazole ring (corresponding to C(6) in the crystal structure) pulls the shared electrons towards the central carbon from both the ring nitrogen atoms, resulting in a number of changes in bond angles and bond lengths. The compound is situated on a general position in the orthorhombic space group *P*2_1_2_1_2_1_. The angle around the C atom, N(2)—C(6)—N(3), is 115.7 (2)°, as compared to 112.2 (2)° in L-histidine, which is consistent with an increase in the *sp*^2^ character at C(6). In addition, angles at N(2) and N(3) are reduced to 104.9 (2)° and 102.7° (as compared to 106.9 (2)°) and 104.9 (2)°), respectively. The bond lengths to N(3) are altered as well, with the bond to C(4) increased to 1.405 (3) Å from 1.382 (2) Å in L-histidine and the bond to C(6) decreased to 1.292 (3) Å from 1.327 (3) Å in L-histidine. The molecule contains an intramolecular hydrogen bond between N(3) of the imidazole side-chain and the amine N(1) with a N–N distance of 2.860 (3) Å. This hydrogen bond is also increased in length from 2.72 Å in L-histidine, again indicative of the electron-withdrawing effect of the fluorine substitution. The structure also contains a number of intermolecular hydrogen bonding interactions: between the carboxylic acid O(2) and the imidazole N(2) of a symmetry related (3/2 - *x*,1 - *y*,-1/2 + *z*) molecule with a O–N distance of 2.741 (3) Å; between the carboxylic acid O(1) and the amine N(1) of a symmetry related (2 - *x*,-1/2 + *y*,1/2 - *z*) molecule with a O–N distance of 2.801 (3) Å; between the carboxylic acid O(2) and the amine N(1) of a symmetry related (1 - *x*,-1/2 + *y*,1/2 - *z*) molecule with a O–N distance of 3.012 (3) Å; and between the carboxylic acid O(1) and the amine N(1) of a symmetry related (1 + *x*,*y*,*z*) molecule with a O–N distance of 2.883 (3) Å.

### Experimental

The compound was synthesized according to a modification of the published procedure (DeClerq, *et al.*, 1978). Trifluoroacetic anhydride was used instead of acetic anhydride to protect the amino group in the first step, which obviates the use of acylase I. In addition, hydrolysis of the *N*-trifluoro acetyl group was carried out with 1 N NaOH in the last step of the synthesis (overall yield, starting from L-histidine methyl ester, is 2.5%). Crystals were grown by slow evaporation of an aqueous solution at room temperature.

### Refinement

Refinement utilized merged data due to the absence of significant anomalous scattering. Hydrogen atoms were included in calculated positions and were not refined.

### Crystal data

**Table:**

C_6_H_8_FN_3_O_2_	*F*(000) = 360
*M**_r_* = 173.15	*D*_x_ = 1.612 Mg m^−^^3^
Orthorhombic, *P*2_1_2_1_2_1_	Mo *K*α radiation, λ = 0.71073 Å
Hall symbol: P 2ac 2ab	Cell parameters from 4008 reflections
*a* = 5.1880 (3) Å	θ = 3.7–20.4°
*b* = 7.3480 (5) Å	µ = 0.14 mm^−^^1^
*c* = 18.7169 (12) Å	*T* = 150 K
*V* = 713.51 (8) Å^3^	Plate, colorless
*Z* = 4	0.16 × 0.14 × 0.13 mm

### Data collection

**Table:**

Bruker APEXII CCD area-detector diffractometer	1352 independent reflections
Radiation source: fine-focus sealed tube	1257 reflections with *I* > 2σ(*I*)
graphite	*R*_int_ = 0.022
phi and ω scans	θ_max_ = 26.0°, θ_min_ = 3.0°
Absorption correction: numerical (*SADABS*; Sheldrick, 2000)	*h* = −6→6
*T*_min_ = 0.978, *T*_max_ = 0.983	*k* = −9→9
3663 measured reflections	*l* = −22→17

### Refinement

**Table:**

Refinement on *F*^2^	Primary atom site location: structure-invariant direct methods
Least-squares matrix: full	Secondary atom site location: difference Fourier map
*R*[*F*^2^ > 2σ(*F*^2^)] = 0.043	Hydrogen site location: inferred from neighbouring sites
*wR*(*F*^2^) = 0.125	H-atom parameters constrained
*S* = 1.06	*w* = 1/[σ^2^(*F*_o_^2^) + (0.0739*P*)^2^ + 0.5836*P*] where *P* = (*F*_o_^2^ + 2*F*_c_^2^)/3
1352 reflections	(Δ/σ)_max_ = 0.035
109 parameters	Δρ_max_ = 0.42 e Å^−^^3^
0 restraints	Δρ_min_ = −0.47 e Å^−^^3^

### Special details

**Table:**

Geometry. All e.s.d.'s (except the e.s.d. in the dihedral angle between two l.s. planes) are estimated using the full covariance matrix. The cell e.s.d.'s are taken into account individually in the estimation of e.s.d.'s in distances, angles and torsion angles; correlations between e.s.d.'s in cell parameters are only used when they are defined by crystal symmetry. An approximate (isotropic) treatment of cell e.s.d.'s is used for estimating e.s.d.'s involving l.s. planes.
Refinement. Refinement of *F*^2^ against ALL reflections. The weighted *R*-factor *wR* and goodness of fit *S* are based on *F*^2^, conventional *R*-factors *R* are based on *F*, with *F* set to zero for negative *F*^2^. The threshold expression of *F*^2^ > σ(*F*^2^) is used only for calculating *R*-factors(gt) *etc*. and is not relevant to the choice of reflections for refinement. *R*-factors based on *F*^2^ are statistically about twice as large as those based on *F*, and *R*- factors based on ALL data will be even larger.

### Fractional atomic coordinates and isotropic or equivalent isotropic displacement parameters (Å^2^)

**Table:**

	*x*	*y*	*z*	*U*_iso_*/*U*_eq_	
C1	0.9310 (5)	0.5230 (3)	0.24487 (13)	0.0145 (5)	
C2	0.8280 (5)	0.5747 (3)	0.31908 (13)	0.0136 (5)	
H2A	0.9601	0.6496	0.3445	0.016*	
C3	0.7728 (6)	0.4010 (4)	0.36301 (13)	0.0173 (6)	
H3A	0.6410	0.3273	0.3380	0.021*	
H3B	0.9322	0.3275	0.3663	0.021*	
C4	0.6798 (5)	0.4441 (3)	0.43659 (14)	0.0162 (5)	
C5	0.7962 (5)	0.4134 (4)	0.50022 (13)	0.0168 (6)	
H5	0.9572	0.3548	0.5077	0.020*	
C6	0.4353 (5)	0.5534 (4)	0.51566 (14)	0.0175 (6)	
F1	0.2479 (3)	0.6321 (2)	0.55168 (9)	0.0310 (5)	
N1	0.5862 (4)	0.6829 (3)	0.31144 (11)	0.0141 (5)	
H1A	0.5249	0.7074	0.2687	0.017*	
H1B	0.5049	0.7222	0.3497	0.017*	
N2	0.6359 (4)	0.4835 (3)	0.55190 (12)	0.0176 (5)	
H2B	0.6594	0.4828	0.5985	0.021*	
N3	0.4443 (4)	0.5346 (3)	0.44706 (12)	0.0175 (5)	
O1	1.1696 (3)	0.4958 (3)	0.24076 (10)	0.0183 (4)	
H1	1.2086	0.4683	0.1986	0.027*	
O2	0.7692 (4)	0.5062 (3)	0.19595 (9)	0.0210 (4)	

### Atomic displacement parameters (Å^2^)

**Table:**

	*U*^11^	*U*^22^	*U*^33^	*U*^12^	*U*^13^	*U*^23^
C1	0.0173 (12)	0.0116 (11)	0.0145 (12)	−0.0012 (11)	0.0038 (11)	−0.0003 (9)
C2	0.0127 (11)	0.0145 (12)	0.0137 (12)	0.0002 (10)	0.0000 (10)	−0.0010 (10)
C3	0.0233 (13)	0.0161 (11)	0.0125 (12)	0.0045 (12)	0.0032 (11)	0.0016 (9)
C4	0.0180 (12)	0.0154 (11)	0.0153 (13)	−0.0001 (10)	0.0029 (10)	0.0027 (10)
C5	0.0173 (13)	0.0156 (12)	0.0176 (13)	−0.0024 (11)	0.0023 (11)	0.0032 (10)
C6	0.0188 (13)	0.0190 (13)	0.0148 (13)	−0.0012 (11)	0.0060 (11)	−0.0003 (10)
F1	0.0305 (9)	0.0356 (10)	0.0269 (9)	0.0061 (8)	0.0057 (8)	−0.0049 (7)
N1	0.0164 (10)	0.0175 (10)	0.0084 (10)	0.0038 (9)	0.0000 (9)	0.0002 (8)
N2	0.0218 (11)	0.0206 (11)	0.0105 (10)	−0.0026 (9)	−0.0013 (8)	0.0004 (9)
N3	0.0186 (10)	0.0185 (10)	0.0156 (11)	0.0032 (10)	0.0011 (9)	0.0009 (9)
O1	0.0167 (9)	0.0227 (9)	0.0155 (9)	−0.0002 (8)	0.0033 (7)	−0.0057 (8)
O2	0.0175 (9)	0.0352 (10)	0.0101 (9)	−0.0026 (9)	−0.0006 (7)	−0.0028 (8)

### Geometric parameters (Å, °)

**Table:**

C1—O2	1.248 (3)	C4—N3	1.405 (3)
C1—O1	1.256 (3)	C5—N2	1.376 (3)
C1—C2	1.536 (4)	C5—H5	0.9500
C2—N1	1.492 (3)	C6—N3	1.292 (3)
C2—C3	1.545 (4)	C6—F1	1.317 (3)
C2—H2A	1.0000	C6—N2	1.344 (4)
C3—C4	1.493 (4)	N1—H1A	0.8800
C3—H3A	0.9900	N1—H1B	0.8800
C3—H3B	0.9900	N2—H2B	0.8800
C4—C5	1.354 (4)	O1—H1	0.8400
			
O2—C1—O1	127.0 (2)	C5—C4—C3	129.2 (2)
O2—C1—C2	117.0 (2)	N3—C4—C3	120.7 (2)
O1—C1—C2	116.0 (2)	C4—C5—N2	106.6 (2)
N1—C2—C1	109.7 (2)	C4—C5—H5	126.7
N1—C2—C3	109.6 (2)	N2—C5—H5	126.7
C1—C2—C3	110.0 (2)	N3—C6—F1	125.6 (2)
N1—C2—H2A	109.2	N3—C6—N2	115.7 (2)
C1—C2—H2A	109.2	F1—C6—N2	118.7 (2)
C3—C2—H2A	109.2	C2—N1—H1A	120.0
C4—C3—C2	112.1 (2)	C2—N1—H1B	120.0
C4—C3—H3A	109.2	H1A—N1—H1B	120.0
C2—C3—H3A	109.2	C6—N2—C5	104.9 (2)
C4—C3—H3B	109.2	C6—N2—H2B	127.6
C2—C3—H3B	109.2	C5—N2—H2B	127.6
H3A—C3—H3B	107.9	C6—N3—C4	102.7 (2)
C5—C4—N3	110.1 (2)	C1—O1—H1	109.5
